# Association Between Discordance of Disease Activity Indices and Quantitative Sensory Testing Measures of Nociplastic Pain in Patients With Rheumatoid Arthritis

**DOI:** 10.1002/acr.25668

**Published:** 2025-11-12

**Authors:** Clarice P. Lin, Burcu Aydemir, Jing Song, Lutfiyya N. Muhammad, Tuhina Neogi, Wendy Marder, Clifton O. Bingham, Marcy B. Bolster, Daniel J. Clauw, Dorothy D. Dunlop, Yvonne C. Lee

**Affiliations:** ^1^ Northwestern University Feinberg School of Medicine Chicago Illinois; ^2^ Boston University Chobanian and Avedisian School of Medicine Boston Massachusetts; ^3^ University of Michigan Ann Arbor; ^4^ Johns Hopkins University School of Medicine Baltimore Maryland; ^5^ Massachusetts General Hospital Boston

## Abstract

**Objective:**

This study investigates the association between discordance in commonly collected clinical indicators of rheumatoid arthritis (RA) disease activity and abnormalities in quantitative sensory testing (QST) observed in individuals with nociplastic pain. The goal is to identify low‐burden methods of assessing nociplastic pain in rheumatology practice.

**Methods:**

Data from 225 patients with active RA were included for cross‐sectional analyses. Measures of discordance in disease activity were as follows: (1) tender–swollen joint count difference (TSJD), (2) proportion of subjective components to the total Disease Activity Score in 28 joints (DAS28‐P), and (3) patient global assessment of disease activity minus evaluator global assessment of Disease Activity (PtGA − EGA). QST measures were pressure pain thresholds (PPTs) at the trapezius, temporal summation (TS), and conditioned pain modulation. We evaluated associations between measures of discordance and QST using unadjusted and multivariable linear regression models.

**Results:**

The mean TSJD was 5.4 (SD ±8.2), and the mean DAS28‐P was 49.7% (SD ±13.3%). The mean PtGA − EGA was 0.7 (SD ±2.2). Higher TSJD was associated with lower trapezius PPT (β = −0.05; 95% confidence interval [CI] −0.08 to −0.02) and higher TS (β = 0.29; 95% CI 0.05 to 0.53). Higher DAS28‐P was associated with a lower trapezius PPT (β = −0.05; 95% CI −0.07 to −0.04) and higher TS (β = 0.21; 95% CI 0.06 to 0.35). PtGA − EGA was not associated with any QST measures.

**Conclusion:**

Two of our proposed measures of discordance (higher TSJD and DAS28‐P) were modestly associated with worse QST measures of nociplastic pain (lower trapezius PPT and higher TS), suggesting that discordance between patient‐reported and physician‐assessed measures of disease activity may reflect an element of nociplastic pain.

## INTRODUCTION

Pain is a predominant symptom of rheumatoid arthritis (RA), with over 71% of patients citing pain as a priority in their care.^1^ Patients with RA may experience nociceptive pain due to active joint inflammation; however, they may also experience other types of pain, which can prove challenging for rheumatology providers to assess and treat.

**SIGNIFICANCE & INNOVATIONS**

- This study analyzed possible associations between discordance in commonly collected clinical indicators of rheumatoid arthritis (RA) disease activity and abnormalities in quantitative sensory testing (QST) observed in individuals with nociplastic pain.
- Modest associations between tender–swollen joint count difference (TSJD), the proportion of subjective components to the total Disease Activity Score in 28 joints (DAS28‐P), and QST measures suggest that TSJD and DAS28‐P could provide information on the presence of nociplastic pain in patients with RA.
- We favor TSJD over DAS28‐P in clinical practice for identifying these patients, given its practicality in application.

Abnormalities in central nervous system pain processing pathways can lead to nociplastic pain, which refers to pain that is not related to tissue lesion or disease or injury.^2^ Nociplastic pain is generally suspected in patients with RA who have pain, despite normalization of markers of inflammation and reduced swollen joint counts.^3^ Structural damage to joints may also contribute to elevated RA indices.^4^ Noninflammatory pain may lead to elevated assessments of RA disease activity, result in overtreatment, and be associated with poor clinical outcomes.^5^ To develop more effective pain management strategies, a deeper comprehension is required to understand noninflammatory pain in patients with RA.

In research settings, quantitative sensory testing (QST) is a method used to assess alterations in nociceptive signaling, which may reflect nociplastic pain.^6^ QST uses different experimental stimuli to elicit and quantitatively measure responses to painful and nonpainful stimuli. QST measures include pressure pain thresholds (PPTs) at the trapezius, temporal summation (TS), and conditioned pain modulation (CPM). Despite their use in research settings, QST measures are challenging to implement in the clinical environment, given the need for rigorous training, time, and controlled settings. These measures have historically been considered the gold standard for diagnosing central sensitization and then nociplastic pain, although these measures are only abnormal in a subset of individuals with conditions such as fibromyalgia.^7^, ^8^, ^9^

Previous studies have proposed several measures of discordance between patient‐reported and physician‐assessed measures of disease activity as potential ways to identify patients with RA and fibromyalgia, the prototypical nociplastic pain condition. Pollard et al found that patients with RA and concomitant fibromyalgia had a significantly larger tender–swollen joint count difference (TSJD) on the standardized 28‐joint examination, compared to those with RA but no fibromyalgia.^10^ McWilliams et al demonstrated that a high proportion of subjective components to the total Disease Activity Score in 28 joints (DAS28‐P), suggests higher levels of nociplastic pain, whereas Salaffi et al suggested that the DAS28‐P showed good discriminatory power in distinguishing patients with RA who have fibromyalgia from those without fibromyalgia.^11^, ^12^ Studenic et al showed that around 80% of the patient global assessment of disease activity (PtGA) is explained by pain, whereas the swollen joint count explained the majority of the evaluator global assessment of disease activity (EGA), thereby suggesting PtGA − EGA as a potential measure for identifying discrepancies between pain and inflammation.^13^

There is a critical need to measure and detect nociplastic pain in patients with RA to decrease pain burden and improve treatment, but there is currently no low‐burden tool used in clinical practice. The components of TSJD, DAS28‐P, and PtGA − EGA are already routinely assessed in clinical practice; however, their associations with research‐based assessments of pain sensitization that may reflect nociplastic pain in patients with RA are unknown. The purpose of this study was to evaluate the relationship between QST measures of pain sensitization with clinical measures of disease discordance. We hypothesized that larger discordance in disease activity measures would be associated with greater evidence of nociplastic pain, defined by lower PPT, higher TS, and lower CPM. If these measures were associated, rheumatology health care providers might be able to incorporate these assessments into their workflow, leading to a better understanding of the impact of nociplastic pain on patients’ experience of disease and, ultimately, improved health outcomes.

## PATIENTS AND METHODS

### Study population

The Central Pain in Rheumatoid Arthritis (CPIRA) study is a prospective, observational cohort study conducted at five academic medical centers, including Brigham and Women's Hospital, Massachusetts General Hospital, University of Michigan Medical Center, Johns Hopkins University, and Boston University School of Medicine.^14^, ^15^ Participants were recruited between 2014 and 2017. Enrollment criteria included the following: (1) aged ≥18 years old, (2) meets American College of Rheumatology (ACR)/EULAR 2010 criteria for RA,^16^ and (3) started on disease‐modifying antirheumatic drugs (DMARDs) or switched DMARDs because of lack of efficacy.^17^ Participants taking centrally acting pain medications were required to be taking stable doses for at least three months before study entry and expected to be taking stable doses for the duration of the study. Patients with known peripheral neuropathy were excluded. Institutional review board approval was obtained at each institution, and all participants provided written informed consent. For the purposes of this specific study, we included 225 participants with complete baseline data in outcome and covariates for cross‐sectional data analysis.

### Clinical assessments and patient‐reported outcomes

Study participants also underwent a standardized assessment of tenderness and swelling at 28 joints by trained assessors. Blood was drawn to assess C‐reactive protein (CRP) level, a measure of inflammation. The DAS28 was calculated using the tender joint count, swollen joint count, PtGA, and CRP level.^11^ The Clinical Disease Activity Index was calculated by adding the 28‐tender joint count, 28‐swollen joint count, PtGA, and EGA.^18^ Smoking status was obtained via self‐report questionnaires administered during clinical visits. Current pain intensity was assessed using a 0‐to‐10 numerical rating scale. Patients also completed the Patient‐Reported Outcomes Measurement Information System (PROMIS) computerized adaptive tests to assess pain interference, depression, and sleep disturbance.^5^, ^19^, ^20^, ^21^ Fibromyalgia was defined according to the ACR 2016 revised fibromyalgia diagnostic criteria.^22^

### QST

QST measures included PPT at the trapezius, TS, and CPM. A brief description of each measure is provided in the following sections. Detailed descriptions have been published previously.^23^

#### PPT at the trapezius

PPTs at the trapezius (a site distant from inflamed joints) reflect overall pain sensitivity. Decreased PPTs at nonarticular sites are thought to be indicative of nociceptive disruption and pain sensitization.^24^ A pressure algometer (Wagner Force 10 FDX) was used to assess PPTs at the center of the trapezius muscle. Pressure was gradually increased until patients indicated that the stimulus was first perceived as painful. Possible PPT values ranged from 0 to 11 kilogram‐force (kgf). Lower values indicate greater pain sensitivity.

#### TS of the forearm

TS is considered a measure of ascending pain facilitation; a high TS is thought to reflect increased responsiveness of the dorsal horn neurons to peripheral stimulation, which is suggestive of nociplastic pain.^25^, ^26^ TS was evaluated by tapping a calibrated probe (MRC Systems) on the patient's forearm. Probes of increasing weight were used to identify the probe that elicited a pain level of 30 to 40 on a 0 to 100 scale. This probe was then tapped 10 times on the forearm. Patients were asked to rate the pain intensity of the first and last stimulus. TS was calculated as the difference between pain intensity at the first and last stimulus. Possible TS values ranged from 0 to 100. Higher values indicate greater pain facilitation.

#### CPM

CPM reflects descending pain inhibition, another proposed mechanism of nociplastic pain. Low CPM suggests decreased activity of the descending analgesic pathways, resulting in inefficient pain inhibition and increased nociplastic pain.^26^, ^27^ To determine CPM, the participant's right hand was immersed in a 5‐ to 7‐°C water bath. The PPT at the left trapezius was measured before and after immersion. CPM was calculated as the ratio of the PPT at the trapezius after immersion to the PPT at the trapezius before immersion. Because CPM is a ratio, the range of possible values is infinite, although typical values range from 0 to 4. Low CPM suggests decreased activity of the descending analgesic pathways, inefficient pain inhibition, and, thus, increased nociplastic pain.

### Discordance measures

The TSJD was calculated by subtracting the 28‐swollen joint count from the 28‐tender joint count.^10^ A positive TSJD indicates a greater number of tender joints relative to swollen joints. A previous study reported that a TSJD ≥7 identifies patients with fibromyalgic RA with 83% sensitivity and 80% specificity.^10^

The DAS28‐P, which is the ratio of subjective components of the DAS28 (tender joint count and patient global assessment) to the total DAS28, was calculated using the following equation^11^:

$$\frac{\left(\left(0.56*\sqrt{\mathrm{TJC}28}+\left(0.014*\text{Patient Global}\right)*10\right)\right)}{\mathrm{DAS}28}*100$$

A previous study reported that a DAS28‐P >63.1% is the optimal cutoff for identifying the presence of fibromyalgia among patients with RA.^11^ The difference between the PtGA and EGA was determined by subtracting the EGA score from the PtGA score.^12^

### Statistical analysis

Descriptive statistics were calculated for demographic and clinical characteristics, including composite disease activity measures. Linear regression models were used to examine relationships between measures of disease discordance and QST measures of pain sensitization suggestive of nociplastic pain. The dependent variables were the QST measures, specifically PPT at the trapezius, TS of the forearm, and CPM. For each QST measure, separate unadjusted and adjusted regression models were conducted for each discordance of disease activity measure, with the independent variables being TSJD, DAS28‐P, and PtGA − EGA. Age, study site, sex, body mass index, RA symptom duration, seropositivity, depression, and sleep disturbance were included as covariates in the adjusted models.

Sensitivity analyses were conducted to explore the effects of fibromyalgia and systemic inflammation. First, to determine if results were impacted by including individuals with fibromyalgia, regression analyses excluding participants who met the ACR 2016 revised fibromyalgia diagnostic criteria were performed. Second, to examine potential confounding by systemic inflammation, we conducted regression models including CRP level as a covariate. All data analyses were performed using R (version 4.5.0). The alpha level was set at *P* < 0.05 for all analyses.

## RESULTS

### Participant characteristics

Of the 225 patients included in this study, the average age was 55.6 years old, with an SD of 13.7. Most were White (77.3%), female (81.3%), and seropositive for rheumatoid factor or anti–cyclic citrullinated peptide (71.6%), with an average symptom duration of 12.4 years (SD 12.6) (Table 1). The mean DAS28 was 4.4 ± 1.2. The mean PROMIS depression t score was 50.2 (SD 9.0), whereas the mean PROMIS sleep disturbance score was 54.4 (SD 9.2), both within population normative levels. Median values for PPT at the trapezius, TS of the forearm, and CPM were 2.6 kgf (interquartile range [IQR] 1.8–3.7), 8.3 (IQR 1.7–20.0), and 1.3 (IQR 1.2–1.5), respectively.

**Table 1:** Characteristics of study participants (n = 225)*

Characteristics	Value
Age, mean ± SD, y	55.6 ± 13.7
Female, n (%)	183 (81.3)
White, n (%)	174 (77.3)
Seropositive, n (%)^a^	161 (71.6)
Body mass index, mean ± SD	28.6 ± 6.9
RA symptom duration, mean ± SD, y	12.4 ± 12.6
Depression, PROMIS t score, mean ± SD	50.2 ± 9.0
Sleep disturbance, PROMIS t score, mean ± SD	54.4 ± 9.2
Fibromyalgia, n (%)	67 (29.8)
Current smoking, n (%)	30 (13.3)
Pain intensity, 0–10 numerical rating scale, mean ± SD	5.2 ± 2.3
Pain interference, PROMIS t score, mean ± SD	60.5 ± 7.3
Oral glucocorticoid use, n (%)	102 (45.3)
Conventional DMARDs, n (%)	92 (40.9)
Biologic/targeted synthetic DMARDs, n (%)	133 (59.1)
Centrally acting pain medications, n (%)	17 (7.6)
Disease Activity Score in 28 joints, mean ± SD	4.4 ± 1.2
Clinical Disease Activity Index, mean ± SD	24.0 ± 14.0
Tender joint count, mean ± SD	10.9 ± 8.6
Swollen joint count, mean ± SD	5.4 ± 5.2
Patient global assessment, mean ± SD	4.2 ± 2.4
Evaluator global assessment, mean ± SD	3.5 ± 2.2
C‐reactive protein, mean ± SD, mg/L	8.0 ± 12.5

* DMARD, disease‐modifying antirheumatic drug; PROMIS, Patient‐Reported Outcomes Measurement Information System; RA, rheumatoid arthritis.

^a^ Seropositive for rheumatoid factor and/or anti–cyclic citrullinated peptide.

**Figure 1:**
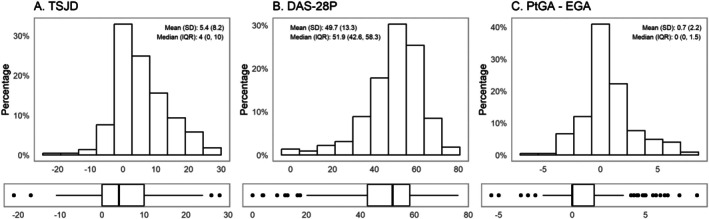
Distribution for disease discordance measures (n = 225). Distribution of disease discordance measures in the Central Pain in Rheumatoid Arthritis cohort, depicted by histogram (with mean and SD) and boxplot (with bold lines indicating median value and edges of boxes corresponding to 25th [lower quartile] and 75th percentiles [upper quartile]). (A) TSJD. (B) DAS28‐P. (C) PtGA − EGA. DAS28‐P, proportion of subjective components of the Disease Activity Score in 28 joints to the total Disease Activity Score in 28 joints; IQR, interquartile range; PtGA − EGA, patient global assessment of disease activity minus evaluator global assessment of disease activity; TSJD, tender–swollen joint count difference.

The mean TSJD was 5.4, with an SD of 8.2. Eighty‐two (36.4%) had a TSJD ≥7 (Supplementary Table 1). The mean DAS28‐P was 49.7%, with an SD of 13.3%. Twenty‐five (11.1%) had a DAS28‐P >63.1%, the previously identified cutoff for fibromyalgia. The mean PtGA ‐ EGA was 0.7 (SD 2.2) (Figure 1).

### Associations between measures of disease discordance and QST measures

In the unadjusted linear regression models (Table 2), higher TSJD was significantly associated with lower PPTs at the trapezius (β = −0.06; 95% confidence interval [CI] −0.08 to −0.03), higher TS of the forearm (β = 0.34; 95% CI 0.12 to 0.56), and higher CPM (β = 0.01; 95% CI 0.002 to 0.01). Higher DAS28‐P was significantly associated with lower PPTs at the trapezius (β = −0.05; 95% CI −0.07 to −0.04) and higher TS of the forearm (β = 0.25; 95% CI 0.11 to 0.38). PtGA − EGA was not significantly associated with PPT at the trapezius, TS of the forearm, or CPM.

**Table 2:** Associations between discordance measures and QST measures from unadjusted and adjusted linear regression models (n = 225)*

Discordance measures	Unadjusted OR (95% CI)	Adjusted OR (95% CI)
Trapezius PPT		
TSJD	**−0.06 (−0.08 to −0.03)**	**−0.05 (−0.08 to −0.02)**
DAS28‐P	**−0.05 (−0.07 to −0.04)**	**−0.05 (−0.07 to −0.04)**
PtGA − EGA	0.08 (−0.18 to 0.02)	0.07 (−0.17 to 0.04)
TS of the forearm		
TSJD	**0.34 (0.12 to 0.56)**	**0.29 (0.05 to 0.53)**
DAS28‐P	**0.25 (0.11 to 0.38)**	**0.21 (0.06 to 0.35)**
PtGA − EGA	0.30 (−0.55 to 1.1)	0.75 (−0.17 to 1.70)
CPM		
TSJD	**0.01 (0.002 to 0.01)**	**0.01 (0.002 to 0.02)**
DAS28‐P	0.003 (−0.0004 to 0.01)	0.003 (−0.001 to 0.01)
PtGA − EGA	0.00 (−0.02 to 0.02)	0.01 (−0.01 to 0.03)

* Models were adjusted for the following covariates: age, study site, sex, body mass index, rheumatoid arthritis symptom duration, seropositivity, depression, and sleep disturbance. Bold values indicate significantly different values (*P* < 0.05). CI, confidence interval, CPM, conditioned pain modulation; DAS28‐P, proportion of subjective components of the Disease Activity Score in 28 joints to the total Disease Activity Score in 28 joints; OR, odds ratio; PPT, pain pressure threshold; PtGA − EGA, patient global assessment of disease activity minus evaluator global assessment of disease activity; QST, quantitative sensory testing; TS, temporal summation; TSJD, tender–swollen joint count difference.

In the adjusted multivariable models (Table 2), higher TSJD remained significantly associated with lower PPTs at the trapezius (β = −0.05; 95% CI −0.08 to −0.02), higher TS of the forearm (β = 0.29; 95% CI 0.05 to 0.53), and higher CPM (β = 0.01; 95% CI 0.002 to 0.02). Higher DAS28‐P was significantly associated with lower PPTs at the trapezius (β = −0.05; 95% CI −0.07 to −0.04) and higher TS of the forearm (β = 0.21; 95% CI 0.06 to 0.35). PtGA − EGA was not significantly associated with PPT at the trapezius, TS of the forearm, or CPM.

### Sensitivity analyses

In sensitivity analyses excluding participants who met the ACR 2016 revised fibromyalgia diagnostic criteria, higher TSJD remained significantly associated with lower PPTs at the trapezius, higher TS at the forearm, and higher CPM (Supplementary Table 2). We also conducted another sensitivity analysis including CRP level as an additional covariate, and findings were consistent with previous results (Supplementary Table 3).

## DISCUSSION

In this cohort of patients with active RA, greater discordances in certain disease activity metrics were associated with worse QST measures of pain sensitization, suggestive of nociplastic pain. However, the strengths of associations were modest, and not all measures of disease discordance were associated with all QST assessments. Consistent patterns included the following: (1) higher TSJD and DAS28‐P were both associated with lower PPT at the trapezius and higher TS, and (2) PtGA − EGA was not significantly associated with any QST measures.

Higher TSJD and DAS28‐P were both consistently associated with lower PPT at the trapezius and higher TS. The consistency in these relationships provides confidence in these results, although the strengths of associations were modest. TSJD and DAS28‐P are composite measures that incorporate the concept of discordance in tender and swollen joint counts in the assessment of nociplastic pain. Tender joint count is similar to PPT (use of a pressure algometer) and TS (use of punctate probes), in that all assess sensitivity to external applications of pressure. Individuals with higher TS are likely to have greater pain sensitivity (lower PPTs) and more tender joints, leading to higher TSJDs and higher DAS28‐P scores.

Conversely, PtGA − EGA was not significantly associated with PPT at the trapezius, TS, or CPM. Neither PtGA nor EGA includes a direct assessment of tender and swollen joint counts. A large observational study previously found EGA to be highly correlated with swollen joint count (ρ > 0.70) and PtGA to be the most highly correlated with patient‐reported pain intensity (ρ > 0.86) and function assessed by the Health Assessment Questionnaire (ρ > 0.49).^13^ Patient‐reported pain intensity and function are important health outcomes that have multiple different causes. Thus, although nociplastic pain may contribute to the discrepancy between PtGA and EGA, there are likely multiple other causes for this discrepancy, such as general health status, mental health, and functional capacity.^28^ In addition, previous studies have suggested that patient education levels, patient health literacy, cultural factors, and evaluator experience may also contribute to these discrepancies.^29^, ^30^, ^31^, ^32^ Lastly, the EGA may be subject to interpretive variability among clinicians. Some assessors may regard it as a measure of inflammatory activity exclusively, in alignment with its application in clinical trial settings, whereas others may integrate a more comprehensive appraisal of disease burden, encompassing pain, fatigue, and functional impairment.^33^

Notably, higher TSJD was weakly associated with higher CPM, but DAS28‐P was not associated with CPM. Although PPT at the trapezius is a reflection of overall central nervous system pain modulation, TS and CPM have been thought to reflect specific pain pathways: ascending pain facilitation and descending pain inhibition, respectively.^34^ Ascending pain facilitation is commonly thought to be stimulated by peripheral input, whereas descending pain inhibition is primarily thought to be determined by genetics and is less likely to change when peripheral nociceptive input is modulated.^35^, ^36^ Given the weak association between TSJD and CPM and the lack of consistency in associations across TSJD and DAS28‐P, we caution against making strong inferences regarding the association (or lack thereof) among TSJD, DAS28‐P, and CPM. It is possible that, in a population of patients with RA and active peripheral joint inflammation, ascending pain facilitation may be the main contributor to pain sensitivity, whereas descending pain inhibition may play a larger role when peripheral inflammation has been treated. It should be noted that even in the prototypical nociplastic condition, fibromyalgia, these QST test results are only abnormal in a subset of patients.^37^, ^38^

Prior studies have suggested various thresholds for tender–swollen joint discrepancies for nociplastic pain. Pollard et al identified a ≥7 TSJD for detecting patients with fibromyalgia RA, with high sensitivity and specificity.^10^ Other studies have suggested that a tender–swollen joint discrepancy as low as 1 may contribute to misrepresentation of RA inflammatory disease activity.^39^, ^40^ Additional research is needed to establish a specific threshold for nociplastic pain using tender and swollen joint counts; however, it is reasonable to conclude that the larger the TSJD, the greater the likelihood of nociplastic pain involvement in patients with RA and chronic pain.^41^ Between TSJD and DAS28‐P, TSJD demonstrates greater feasibility for implementation in a clinical setting because of its ease of basic calculation and assessment. Use of TSJD in clinical practice may identify patients who would benefit from stronger reliance on exercise, psychological treatment, and noninflammatory pharmacologic treatment and less focus on DMARDs alone for pain management.

Study strengths include the large sample size as well as involvement of multiple centers, which increases the generalizability of our results. To our knowledge, the CPIRA is the largest cohort of patients with RA providing comprehensive data on multiple QST measures, including PPT at the trapezius, TS, and CPM. These QST measures have also been previously demonstrated to be associated with pain intensity and treatment response in patients with RA.^17^, ^42^ This study also sought to reduce the heterogeneity of the QST evaluators by use of standardized QST protocol and rigorous training standards.^23^

Limitations of this study include that this was a cross‐sectional analysis of patients with active RA, so results are only specific to this disease state. The cross‐sectional analysis also precludes causal inferences. Another limitation is that, although QST measures are commonly used to assess nociplastic pain in research studies, there is no gold standard for assessing nociplastic pain, and QST tests are not consistently different in individuals with nociplastic pain (eg, individuals with fibromyalgia) than in healthy controls.^37^, ^38^ In addition, a further limitation is the lack of objective and sensitive assessment of synovitis, such as ultrasonography or magnetic resonance imaging, which could have enabled a more accurate characterization of joint inflammation. Moreover, the inclusion of patients taking centrally acting neuropathic pain medications, such as duloxetine or pregabalin, may influence pain processing. However, we could not evaluate this rigorously because only 7.6% of the sample were taking centrally acting medications.

In summary, discordances in disease activity metrics (eg, TSJD and DAS28‐P) are useful to consider as potential indicators of nociplastic pain in the clinical setting. By identifying or excluding nociplastic pain as a potential contributor to patient symptoms, health care providers can provide more individualized and targeted recommendations about pain management. This could range from recommending intensification of immunosuppressive therapy to treat inflammatory pain to recommending the addition of a tricyclic antidepressant or serotonin–norepinephrine reuptake inhibitor for nociplastic pain. We would favor TSJD over DAS28‐P in clinical practice for identifying these patients, given its ease of calculation. Although our analysis focused on routinely collected clinical measures such as TSJD, DAS28‐P, and PtGA − EGA, we acknowledge that other patient‐reported measures may present potential practical and reliable alternatives. Future research should investigate the comparative utility of these self‐report instruments, given their feasibility and applicability in routine rheumatologic practice.

## AUTHOR CONTRIBUTIONS

All authors contributed to at least one of the following manuscript preparation roles: conceptualization and/or methodology, software, investigation, formal analysis, data curation, visualization, and validation and drafting or reviewing/editing the final draft. As corresponding author, Dr Lin confirms that all authors have provided the final approval of the version to be published and takes responsibility for the affirmations regarding article submission (eg, not under consideration by another journal), the integrity of the data presented, and the statements regarding compliance with institutional review board/Declaration of Helsinki requirements.

## Supporting information


**Disclosure Form**:

**Supplementary Table 1** Clinical Disease Activity Scores Stratified by TSJD ≥ 7 vs. < 7 and DAS28‐P > 63.1% vs. ≤ 63.1%
**Supplementary Table 2**. Associations Between Discordance Measures and QST Measures From Unadjusted and Adjusted Linear Regression Models, Restricting to Patients Without Fibromyalgia (n = 158)
**Supplementary Table 3**: Associations Between Discordance Measures and QST Measures From Adjusted Linear Regression Models with CRP as a Covariate (n = 225)
