# Supplementary material for: Association Between Discordance of Disease Activity Indices and Quantitative Sensory Testing Measures of Nociplastic Pain in Patients With Rheumatoid Arthritis
Source: Arthritis Care Res (Hoboken). 2025 Nov 12;78(1):47–53. doi: 10.1002/acr.25668 (PMC12752918; doi:10.1002/acr.25668)
Supplement: Supplementary file 2 — Supplementary Table 1 Clinical Disease Activity Scores Stratified by TSJD ≥ 7 vs. < 7 and DAS28‐P > 63.1% vs. ≤ 63.1% Supplementary Table 2. Associations Between Discordance Measures and QST Measures From Unadjusted and Adjusted Linear Regression Models, Restricting to Patients Without Fibromyalgia (n = 158) Supplementary Table 3: Associations Between Discordance Measures and QST Measures From Adjusted Linear Regression Models with CRP as a Covariate (n = 225) [file ACR-78-47-s001.docx]

**SUPPLEMENTARY INFORMATION:**

**Supplementary Table 1: Clinical Disease Activity Scores Stratified by TSJD ≥ 7 vs. < 7 and DAS28-P > 63.1% vs. ≤ 63.1%**

| **Clinical Disease Activity Scores** | **FM Defined by TSJD** | | **FM Defined by DAS28-P** | |
| --- | --- | --- | --- | --- |
|  | **TSJD ≥ 7**  **(n = 82)** | **TSJD < 7**  **(n = 143)** | **DAS28-P > 63.1% (n = 25)** | **DAS28-P ≤ 63.1% (n = 200)** |
| DAS28 | 5.2 ± 0.9 | 3.9 ± 1.2 | 4.6 ± 0.9 | 4.3 ± 1.3 |
| CDAI | 33.2 ± 11.2 | 18.8 ± 12.7 | 31.1 ± 10.9 | 23.2 ± 14.1 |
| Tender Joint Count | 19.1 ± 6.4 | 6.2 ± 5.6 | 18.8 ± 7.5 | 9.9 ± 8.2 |
| Swollen Joint Count | 5.0 ± 4.3 | 5.7 ± 5.6 | 2.2 ± 2.7 | 5.9 ± 5.3 |
| Patient Global Assessment | 4.8 ± 2.2 | 3.9 ± 2.5 | 5.6 ± 2.5 | 4.0 ± 2.4 |
| Evaluator Global Assessment | 4.4 ± 2.1 | 3.0 ± 2.2 | 4.5 ± 2.5 | 3.4 ± 2.2 |
| CRP (mg/L) | 7.4 ± 9.2 | 8.4 ± 14.0 | 1.1 ± 1.0 | 8.9 ± 12.9 |

* Values are means ± standard deviation

**Supplementary Table 2. Associations Between Discordance Measures and QST Measures From Unadjusted and Adjusted Linear Regression Models, Restricting to Patients Without Fibromyalgia (n = 158)**

| **Dependent Variable: Pain Pressure Threshold of Trapezius (PPT)** | | |
| --- | --- | --- |
| **Discordance Measures** | Unadjusted Beta [95% CI] | Adjusted* Beta [95% CI] |
| TSJD | **-0.05 [-0.09, -0.02]** | **-0.05 [-0.09, -0.01]** |
| DAS28-P | **-0.05 [-0.07, -0.04]** | **-0.05 [-0.07, -0.04]** |
| PtGA - EGA | -0.12 [-0.24, 0.01] | -0.12 [-0.25, 0.02] |
| **Dependent Variable: Temporal Summation of Forearm (TS)** | | |
| **Discordance Measures** | Unadjusted Beta [95% CI] | Adjusted* Beta [95% CI] |
| TSJD | **0.31 [0.04, 0.58]** | **0.36 [0.04, 0.67]** |
| DAS28-P | **0.23 [0.08, 0.38]** | **0.24 [0.08, 0.40]** |
| PtGA - EGA | 0.28 [-0.76, 1.30] | 0.68 [-0.45, 1.80] |
| **Dependent Variable: Conditioned Pain Modulation (CPM)** | | |
| **Discordance Measures** | Unadjusted Beta [95% CI] | Adjusted* Beta [95% CI] |
| TSJD | **0.01 [0.001, 0.01]** | **0.01 [0.001, 0.02]** |
| DAS28-P | 0.003 [-0.001, 0.01] | 0.003 [-0.001, 0.01] |
| PtGA - EGA | -0.01 [-0.04, 0.02] | 0.01 [-0.02, 0.03] |

*Models adjusted for age, study site, sex, BMI, symptom duration, seropositivity, depression, and sleep disturbance

TSJD = Tender minus swollen joint count

DAS28-P = Ratio of subjective components of DAS-28 over total DAS-28

PtGA – EGA = Patient global assessment minus evaluator global assessment

**Supplementary Table 3: Associations Between Discordance Measures and QST Measures From Adjusted Linear Regression Models with CRP as a Covariate (n = 225)**

| **Discordance Measures** | **QST Measures (Adjusted Beta [95% CI])** | |
| --- | --- | --- |
|  | Pain Pressure Threshold of Trapezius (PPT) | Temporal Summation of Forearm (TS) |
| TSJD | **-0.05 [-0.08, -0.03]** | **0.31 [0.06, 0.55]** |
| DAS28-P | **-0.06 [-0.07, -0.04]** | **0.25 [0.10, 0.40]** |

Models adjusted for covariates age, study site, sex, BMI, symptom duration, seropositivity, depression, sleep disturbance, and CRP

TSJD = Tender swollen joint count difference

DAS28-P = Ratio of subjective components of DAS-28 over total DAS-28
